# Triterpenoids and Sterols from the Leaves and Twigs of *Melia azedarach*

**DOI:** 10.1007/s13659-014-0019-1

**Published:** 2014-05-09

**Authors:** Wei-Ming Zhang, Jie-Qing Liu, Xing-Rong Peng, Luo-Sheng Wan, Zhi-Run Zhang, Zhong-Rong Li, Ming-Hua Qiu

**Affiliations:** 1 State Key Laboratory of Phytochemistry and Plant Resources in West China, Kunming Institute of Botany, Chinese Academy of Sciences, Kunming, 650201 China; 2 University of Chinese Academy of Sciences, Beijing, 100049 China

**Keywords:** Meliaceae, *Melia azedarach*, Triterpenoids, Sterols

## Abstract

Two new triterpenoids (**1** and **2**) and a new sterol (**3**), together with six known constituents (**4**–**9**), were isolated from the leaves and twigs of *Melia azedarach*. Their chemical structures were elucidated on the basis of spectroscopic analysis.

**Electronic supplementary material:**

The online version of this article (doi:10.1007/s13659-014-0019-1) contains supplementary material, which is available to authorized users.

## Introduction

*Melia azedarach* Linn. (Meliaceae) are widely distributed in southern districts of the Yellow River in China. The fruits and bark are commonly used as famous Traditional Chinese Medicine for acesodyne and disinsection [1]. This species has been reported to contain triterpenoids, steroids, limonoids, flavonoid glycosides, and simple phenolics [2], which have been found to possess some benefic pharmacological effects, including analgesic, anticancer, antiviral, antimalarial, antibacterial, and antifeedant activities [3, 4].

**Fig. 1:**
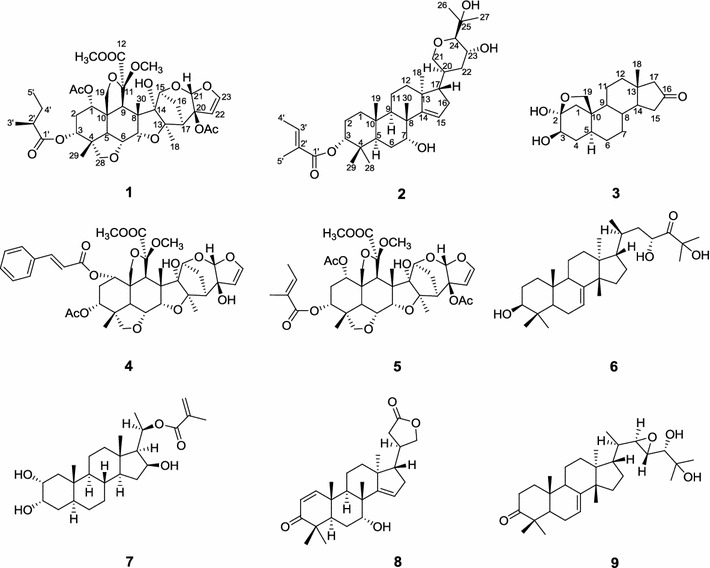
The structures of compounds **1**–**9**

As a well known natural pesticide, azadirachtin has attracted much attention [5]. Previous investigations of the bark and roots of *M. azedarach* have shown that it is a rich source of meliacarpinin type limonoids [6–10]. Until now, few chemical studies have analyzed its leaves and twigs, which prompted us to conduct this project. We identified three new compounds: a meliacarpinin type limonoid (**1**), an apotirucallane derivative (**2**), and a sterol (**3**), together with six known compounds (**4**–**9**) (Fig. 1). Herein, we report the details of the isolation, structural elucidation of compounds **1**–**3**.

## Results and Discussion

The air-dried powder of *M. azedarach* leaves and twigs was extracted with MeOH (30 L × 3) at room temperature three times to give the residue, which was then partitioned between CHCl_3_ and water to get the CHCl_3_ soluble fraction. Then, three new constituents together with six known compounds were acquired by a series of chromatographic methods. Herein, we described the isolation and structural elucidation of these new compounds.

**Fig. 2:**
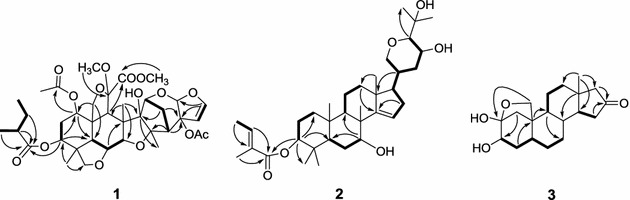
Selected ^1^H-^1^H COSY () and HMBC () correlations of **1**–**3**

Compound **1** was isolated as an amorphous powder. The molecular formula was determined as C_37_H_50_O_15_ from the HREIMS ion peak at *m*/*z* 734.3159 [M]^+^ (calcd for 734.3150). Its IR spectrum showed the presence of hydroxyl (3456 cm^−1^) and carbonyl (1739 cm^−1^) groups. The 1D NMR data (Table 1) of **1** displayed characteristic signals of meliacarpinin skeleton with three methyls (*δ*_H_ 1.75, s, 3H; *δ*_H_ 0.95, s, 3H; *δ*_H_ 1.66, s, 3H), two methoxyls (*δ*_H_ 3.29, s, 3H; *δ*_H_ 3.79, s, 3H), two acetyls (*δ*_H_ 1.90, s, 3H; *δ*_H_ 2.30, s, 3H), one 2-methylbutyryl (*δ*_H_ 2.59, m; *δ*_H_ 1.27, d, *J* = 7.1 Hz; *δ*_H_ 1.53, m; *δ*_H_ 2.02, m; *δ*_H_ 0.99, t, *J* = 7.4 Hz) and one hydroxyl (*δ*_H_ 4.34, s, 1H) groups, which had a close resemblance to 3-tigloyl-1,20-diacetyl-11-methoxymeliacarpinin [8], except for the presence of one 2-methylbutyryl moiety in **1** instead of the tigloyl group at C-3 in. 3-tigloyl-1,20-acetyl-11-methoxymeliacarpinin. Observed the HMBC correlations (Fig. 2) of of H-2′ (*δ*_H_ 2.59, m), H-3′ (*δ*_H_ 1.27, d, *J* = 7.1 Hz), H-4′a (*δ*_H_ 1.53, m) with C-1′ (*δ*_C_ 176.1), and ^1^H-^1^H COSY correlations of H-3′/H-2′/H-4′/H-5′ (*δ*_H_, 0.99, t, *J* = 7.4 Hz) confirmed above deduction. The linkage of 2-methylbutyryl moiety to C-3 was determined by the HMBC correlations from H-3 (*δ*_H_ 4.96, br. t, *J* = 2.7 Hz) to C-1 (*δ*_C_ 71.2), C-5 (*δ*_C_ 35.2), and C-1′.

**Table 1:** ^1^H NMR and ^13^C NMR spectroscopic data of **1** and **2**

Pos	**1** ^a^		Pos	**2** ^b^	
	*δ*_H_ (*J*, Hz)	*δ*_C_		*δ*_H_ (*J*, Hz)	*δ*_C_
1	4.26 (d, 9.3)	71.2 d	1a	1.27 (m)	35.0 t
2a	2.27 (m)	28.4 t	1b	1.43 (m)	
2b	2.34 (m)		2a	1.60 (m)	23.9 t
3	4.96 (br. t, 2.7)	71.6 d	2b	1.99 (m)	
4		42.9 s	3	4.65 (t, 2.7)	80.1 d
5	3.33 (d, 12.7)	35.2 d	4		37.7 s
6	4.12 (br. d, 9.2)	72.1 d	5	2.09 (m)	43.5 d
7	4.53 (br. d, 5.7)	84.0 d	6a	1.71 (m)	25.6 t
8		52.3 s	6b	1.83 (m)	
9	3.84 (s)	48.5 d	7	3.95 (s-like)	74.1 d
10		50.1 s	8		45.3 s
11		107.7 s	9	2.12 (m)	43.7 d
12		170.5 s	10		38.9 s
13		94.1 s	11a	1.53 (m)	17.9 t
14		93.2 s	11b	1.71 (m)	
15	4.34 (overlap)	82.3 d	12a	1.55 (m)	36.3 t
16a	1.93 (m)	29.4 t	12b	1.93 (m)	
16b	2.26 (m)		13		47.9 s
17	3.18 (d, 5.9)	48.7 d	14		162.7 s
18	1.75 (s)	26.2 q	15	5.49 (d, 2.4)	121.1 d
19a	4.12 (br. d, 9.2)	70.7 t	16a	2.12 (m)	35.9 t
19b	5.01 (overlap)		16b	2.31 (ddd, 15.1, 7.3, 3.6)	
20		92.2 s	17	2.04 (m)	53.8 d
21	5.98 (s)	106.7 d	18	1.03 (s)	19.6 q
22	5.59 (d, 3.0)	106.2 d	19	0.96 (s)	16.1 q
23	6.65 (d, 3.0)	147.6 d	20	1.94 (m)	37.4 d
28a	3.68 (d, 3.0)	76.7 t	21a	3.46 (dd, 11.5, 2.6)	71.3 t
28b	3.70 (br. s)		21b	4.02 (d, 11.5)	
29	0.95 (s)	18.2 q	22a	2.01 (m)	37.6 t
30	1.66 (s)	18.5 q	22b	1.56 (m)	
14-OH	4.34 (s)		23	3.83 (ddd, 10.8, 9.0, 4.6)	65.7 d
11-OMe	3.29 (s)	52.4 q	24	2.88 (d, 9.0)	87.8 d
12-OMe	3.79 (s)	53.0 q	25		74.5 s
1-CH_3_CO		170.5 s	26	1.22 (s)	24.6 q
20-CH_3_CO		171.2 s	27	1.23 (s)	28.0 q
1-CH_3_COO	1.90 (s)	20.6 q	28	0.85 (s)	28.4 q
20-CH_3_COO	2.30 (s)	21.5 q	29	0.95 (s)	22.4 q
1′		176.1 s	30	1.11 (s)	28.7 q
2′	2.59 (m)	41.0 d	1′		169.3 s
3′	1.27 (d, 7.1)	16.7 q	2′		130.3 s
4′a	1.53 (m)	26.3 t	3′	6.92 (qq, 7.1, 1.4)	138.6 d
4′b	2.02 (m)		4′	1.81 (dd, 7.1, 1.1)	14.6 q
5′	0.99 (t, 7.4)	11.8 q	5′	1.85 (s-like)	12.4 q

^a^Recorded in C_5_D_5_N; ^1^H and ^13^C NMR recorded at 500, 125 MHz

^b^Recorded in CD_3_OD; ^1^H and ^13^C NMR recorded at 600, 150 MHz

**Fig. 3:**
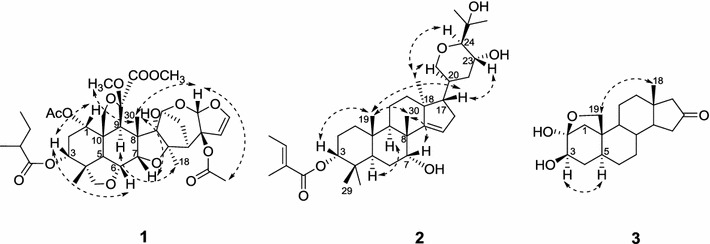
Selected ROESY () correlations of **1**–**3**

The absolute configuration of C-2′ was determined as *S,* supported by the [*α*]_D_^15^ value at +16.3 of (*S*)-2-methylbutyric acid derived from **1** by alkaline hydrolysis ([*α*]_D_^22^ −14.3 for (*R*)-2-methylbutyric acid and [*α*]_D_^25^ +19.3 for (*S*)-2-methylbutyric acid) [11, 12]. The ROESY correlation (Fig. 3) between H-3 and H-6*β* (*δ*_H_ 4.12, br. d, *J* = 9.2 Hz) indicated that the 2-methylbutyryloxy was *α*-oriented. Other relative configuration of **1** were identical with those of 3-tigloyl-1,20-acetyl-11-methoxyneliacarpinin on the basis of ROESY spectrum. Therefore, chemical structure of **1** was deduced as 3*α*-(2-methylbutyryl)- 1,20-diacetyl-11-methoxymeliacarpinin.

Compound **2** was obtained as an amorphous powder. Based on the positive HREIMS (*m*/*z* 572.4083, calcd for 572.4077), the molecular formula was defined as C_35_H_56_O_6_. The ^1^H NMR, ^13^C-DEPT (Table 1) spectra showed the presence of nine methyls (two of which belonged to a tigloyl), eight methylenes (one oxygenated), eight methines (four oxygenated), one trisubstituted double bond, and four quaternary carbon. These data suggested that **2** was the apo-tirucallol (euphol) skeleton [13]. Comparison of NMR data of **2** with those of compound **5** (CAS NO: 1002345-41-6) revealed that they were similar [14], except that a senecioyl ester side chain at C-3 in compound **5** was replaced by a tigloyl group (*δ*_C_ 169.3 C-1′, 130.3 C-2′, 138.6 C-3′, 14.6 C-4′, and 12.4 C-5′) in **2** [8], which was confirmed by the HMBC correlations (Fig. 2) of H-3 (*δ*_H_ 4.65, t, *J* = 2.7 Hz), H-3′ (*δ*_H_ 6.92, qq, *J* = 7.1, 1.4 Hz), and H-5′ (*δ*_H_ 1.85, s-like) with C-1′, and of H-4′ (*δ*_H_ 1.81, dd, *J* = 7.1, 1.1 Hz) with C-2′, together with the ^1^H-^1^H COSY correlations of H-3′/H-4′.

The ROESY correlation (Fig. 3) between H-3 and Me-19*β* suggested that the tigloyl group at C-3 was *α*-oriented. The coupling constant between H-23 and H-24 (*J* = 9.0 Hz) suggested their anti-periplanar relation [14], and combination with the ROESY correlations of H-17/H-23, H-17/H-19*β*, H-20/Me-18*α* and H-24/Me-18*α* revealed that the configuration of C-23 and C-24 were both R*. Thus, the structure of **2** was established as 3*α*-tigloyl-17*α*-20*S*-21,24-epoxy-apotirucall-14-en-7*α*,23*α*,25-triol.

Compound **3** was isolated as an amorphous powder. The HREIMS of **3** gave a [M]^+^ ion peak at *m*/*z* 320.1985 (calcd for 320.1988), consistent with the molecular formula of C_19_H_28_O_4_. Detailed analysis of its ^1^H and ^13^C-DEPT (Table 2) and 2D NMR data indicated that **3** and 2*α*,3*α*-dihydroxyandrostan-16-one 2*β*,19-hemiketal [15] had the same planar structure. The only difference between them was the configuration of substituent group at C-3. Comparison its ^1^H NMR data with that of *epi*-isomer showed that the coupling constants of H-3 (*δ*_H_ 4.11, dd, *J* = 10.3, 6.0 Hz) and the chemical shifts for H-1*α* (*δ*_H_ 1.38, d, *J* = 11.3 Hz) and H-1*β* (*δ*_H_ 2.54, d, *J* = 11.3 Hz) were obviously different from those of 2*α*,3*α*-dihydroxyandrostan-16-one 2*β*,19-hemiketa. But the aforementioned data was familiar with 2*α*,3*β*-dihydroxypregnan-16-one 2*β*,19-hemiketal [10], which implied that the H-3 of **3** was *α*-oriented. This conclusion further confirmed by the cross peak between H-3 and H-5 (*δ*_H_ 1.38, overlap) in the ROESY spectrum (Fig. 3). So the hydroxyl group at C-3 was *β*-configuration. Consequently, the chemical structure of **3** was elucidated as 2*α*,3*β*-dihydroxyandrostan-16-one 2*β*,19-hemiketal.

**Table 2:** ^1^H NMR and ^13^C NMR spectroscopic data of **3**

Pos	*δ*_H_ (*J*, Hz)	*δ*_C_	Pos	*δ*_H_ (*J*, Hz)	*δ*_C_
1a	1.38 (d, 11.3)	44.3 t	11a	1.34 (m)	21.5 t
1b	2.54 (d, 11.3)		11b	1.58 (m)	
2		106.3 s	12a	1.20 (m)	38.2 t
3	4.11 (dd, 10.3, 6.0)	74.7 d	12b	1.59 (m)	
4a	1.73 (m)	39.1 t	13		39.2 s
4b	2.19 (m)		14	1.24 (m)	51.7 d
5	1.38 (overlap)	43.8 d	15a	1.84 (m)	39.7 t
6a	1.16 (m)	29.8 t	15b	2.14 (dd, 17.9, 7.5)	
6b	1.46 (m)		16		217.5 s
7a	0.79 (overlap)	32.3 t	17a	1.93 (d, 16.6)	56.2 t
7b	1.37 (overlap)		17b	2.06 (d, 16.6)	
8	0.80 (overlap)	36.8 d	18	0.64 (s)	18.1 q
9	1.05 (m)	46.4 d	19a	3.86 (d, 8.1)	67.6 t
10		48.2 s	19b	4.08 (d, 8.1)	

Recorded in C_5_D_5_N; ^1^H and ^13^C NMR recorded at 600, 150 MHz

Six known constituents: 1-cinnamoyl-3-acetyl-11-methoxymeliacarpinin (**4**) [8], 3-tigloyl-1,20-diacetyl-11-methoxymeliacarpinin (**5**) [8], 3*S*,23*R*,25-trihydroxytirucall-7-en-24-one (**6**) [16], and 2*α*,3*α*,16*β*-trihydroxy-5*α*-pregnane 20*R*-methacrylate (**7**) [17], 6-de(acetyloxy)-7-deacetylchisocheton compound E (**8**) [18], Toonapubesin C (**9**) [19], were identified by comparison of their spectroscopic data with those reported in the literature.

## Experimental

### General Experimental Procedures

Optical rotations were measured with a Horiba SEPA-300 polarimeter. UV spectra were detected on a Shimadzu UV-2401A spectrophotometer. IR spectra were measured on a Bruker Tensor-27 infrared spectrophotometer with KBr pellets. ESIMS analysis were recorded on an API QSTAR Pulsar I spectrometer. EIMS and HREIMS were performed on a Waters Autospec Premier P776 mass spectrometer. 1D and 2D NMR spectra were recorded on Bruker DRX-500 and Bruker Avance III-600 spectrometers with TMS as internal standard. Semi-preparative HPLC studies were carried out on an Agilent 1100 liquid chromatograph with a Zorbax SB-C18 (9.4 mm × 25 cm) column. Column chromatography was performed with silica gel (200–300 mesh, Qingdao Marine Chemical, Inc.), Sephadex LH-20 (20–150 μm, Pharmacia), and Lichroprep RP-18 (40**–**63 μm, Merck). Fractions were monitored by TLC, and spots were visualized by heating the silica gel plates sprayed with 10 % H_2_SO_4_ in EtOH.

### Plant Material

The leaves and twigs of *M. azedarach* were collected from Kunming, Yunnan Province, China. A voucher sample (NO: 2011-05-07) has been deposited in the State Key Laboratory of Phytochemistry and Plant Resources in West China, Kunming Institute of Botany, Chinese Academy of Sciences.

### Extraction and Isolation

The air-dried and powdered leaves and twigs of *M. azedarach* (10 kg) were extracted with MeOH (30 L × 3) at room temperature. Evaporation of the solvent under reduced pressure provide a dark residue (700 g), which was suspended in water and then partitioned with CHCl_3_ and *n*-BuOH, successively, to yield CHCl_3_ fraction (120 g), *n*-BuOH fraction (156 g). The CHCl_3_ extract was chromatographed by silica gel column eluted with CHCl_3_-MeOH as a gradient (100:1, 50:1, 20:1, 5:1) to afford four fractions. The CHCl_3_-MeOH (100:1) portion was evaporated to obtain a residue (20 g), which was subjected to silica gel chromatograph column with petroleum ether-EtOAc (10:1, 6:1, 3:1, 1:1) as elution, to give four fractions (A, B, C, and D). Fraction B (5 g) was further subjected to RP-18 chromatograph column, eluting with MeOH-H_2_O (40:60, 60:40, 80:20, and 100:0) to afford five fractions: B1–B5. Fraction B4 was then purified by HPLC (70 % CH_3_CN aq.; 2.0 mL/min; 210 nm; Zorbax SB-C18, 9.4 mm × 25 cm) to give compounds **1** (4 mg), **4** (2 mg) and **5** (3 mg). In the same way, **2** (4 mg), **6** (5 mg) and **9** (7 mg) were islated from fraction B3. Fraction B2 was subjected to silica gel chromatograph column with petroleum ether-EtOAc (8:1, 5:1, 3:1, 1:1, and 0:1) as elution, to give five subfractions (E, F, G, and H). Subfraction F was further separated and purified by silica gel chromatography column with CHCl_3_-Me_2_CO (50:1, 20:1, 5:1, and 1:1) as elution, get four subfraction: F1–F4, subfraction F2 was successively subjected to Sephadex LH-20 (MeOH) and HPLC (80 % CH_3_CN aq.; 2.0 mL/min; 210 nm; Zorbax SB-C18, 9.4 mm × 25 cm), and compounds **3** (1.5 mg), **7** (3 mg) and **8** (6 mg) were obtained.

### 3*α*-(2-Methylbutyryl)-1,20-diacetyl-11-methoxymeliacarpinin (**1**)

Amorphous powder; [*α*]_D_^17^ –17.8 (*c* 0.08, MeOH); UV (MeOH) λ_max_ (log *ε*) 208 (4.09) nm; IR (KBr) ν_max_ 3456, 2953, 1739, 1706, 1618, 1438, 1376, 1252, 1160, 1131, 1061, and 949 cm^−1^; ^1^H NMR (500 MHz, C_5_D_5_N) and ^13^C DEPT (125 MHz, C_5_D_5_N) data, see Tables 1 and 2; positive ESIMS *m*/*z* 757 [M+Na]^+^; positive HREIMS *m*/*z* 734.3159 (calcd for C_37_H_50_O_15_ [M]^+^, 734.3150).

### 3*α*-Tigloyl-17*α*-20*S*-21,24-epoxy-apotirucall-14-en-7*α*,23*α*,25-triol (**2**)

Amorphous powder; [*α*]_D_^17^ –28.9 (*c* 0.20, MeOH); UV (MeOH) λ_max_ (log *ε*) 204 (3.80) nm; IR (KBr) ν_max_ 3441, 2927, 2855, 1631, 1452, 1384, 1268, 1075 and 578 cm^−1^; ^1^H NMR (600 MHz, CD_3_OD) and ^13^C DEPT (150 MHz, CD_3_OD) data, see Tables 1 and 2; positive ESIMS *m*/*z* 595 [M+Na]^+^; positive HREIMS *m*/*z* 572.4083 (calcd for C_35_H_56_O_6_ [M]^+^, 572.4077).

### 2*α*,3*β*-Dihydroxyandrostan-16-one 2*β*,19-hemiketal (**3**)

Amorphous powder; [*α*]_D_^17^ –48.0 (*c* 0.30, MeOH); UV (MeOH) λ_max_ (log *ε*) 202 (3.56), 219 (3.51) nm; IR (KBr) ν_max_ 3464, 2924, 2874, 1720, 1447, 1295, 1187, 1130, 1044, and 993 cm^−1^; ^1^H NMR (600 MHz, C_5_D_5_N) and ^13^C DEPT (150 MHz, C_5_D_5_N) data, see Tables 1 and 2; positive ESIMS *m*/*z* 343 [M+Na]^+^; positive HREIMS *m*/*z* 320.1985 (calcd for C_20_H_28_O_5_ [M]^+^, 320.1988).

## Electronic supplementary material

Below is the link to the electronic supplementary material.

Supplementary material 1 (DOC 1115 kb)
