# Supplementary material for: Triterpenoids and Sterols from the Leaves and Twigs of Melia azedarach
Source: Nat Prod Bioprospect. 2014 May 9;4(3):157–62. doi: 10.1007/s13659-014-0019-1 (PMC4050312; doi:10.1007/s13659-014-0019-1)
Supplement: Supplementary file 1 — Supplementary material 1 (DOC 1115 kb) [file 13659_2014_19_MOESM1_ESM.doc]

Supporting Information Available

**Triterpenoids and** **Sterols from the Leaves and Twigs of**

***Melia azedarach***

Wei-ming Zhang,^a,b^ Jie-qing Liu,^a^ Xing-rong Peng,^a^ Luo-sheng Wan,^a^ Zhi-run Zhang,^a^ Zhong-rong Li,^a^ Ming-hua Qiu^a,b^,*

^a^State Key Laboratory of Phytochemistry and Plant Resources in West China, Kunming Institute of Botany, Chinese Academy of Sciences, Kunming 650201, China

^b^University of Chinese Academy of Sciences, Beijing 100049, China

**Correspondence**

***MingHua Qiu***

State Key Laboratory of Phytochemistry and Plant Resources in West China

Kunming Institute of Botany, Chinese Academy of Sciences

132 LanHei Road

Kunming 650201, Yunnan, P. R. China.

Tel: +86-871-65223327

Fax: +86-871-65223255

Email address: [mhchiu@mail.kib.ac.cn](mailto:mhchiu@mail.kib.ac.cn)

**S 1.** ^1^H NMR spectrum of compound **1** (C_5_D_5_N, 500 MHz).

**S 2.** ^13^C NMR spectrum of compound **1** (C_5_D_5_N, 125 MHz).

**S 3.** HMBC spectrum of compound **1**.

**S 4.** COSY spectrum of compound **1**.

**S 5.** HSQC spectrum of compound **1**.

**S 6.** ROESY spectrum of compound **1**.

**S 7.** ^1^H NMR spectrum of compound **2** (CD_3_OD, 600 MHz).

**S 8.** ^13^C NMR spectrum of compound **2** (CD_3_OD, 150 MHz).

**S 9.** HMBC spectrum of compound **2**.

**S 10.** COSY spectrum of compound **2**.

**S 11.** HSQC spectrum of compound **2**.

**S 12.** ROESY spectrum of compound **2**.

**S 13.** ^1^H NMR spectrum of compound **3** (C_5_D_5_N, 600 MHz).

**S 14.** ^13^C NMR spectrum of compound **3** (C_5_D_5_N, 150 MHz).

**S 15.** HMBC spectrum of compound **3**.

**S 16.** COSY spectrum of compound **3**.

**S 17.** HSQC spectrum of compound **3**.

**S 18.** ROESY spectrum of compound **3**.

Compound **1**

**S 1.** ^1^H NMR spectrum of compound **1** (C_5_D_5_N, 500 MHz).

**S 2.** ^13^C NMR spectrum of compound **1** (methanol-*d*_4_ and chloroform-*d*, 150 MHz).

**S 3.** HMBC spectrum of compound **1**.

**S 4.** COSY spectrum of compound **1**.

**S 5.** HSQC spectrum of compound **1**.

**S 6.** ROESY spectrum of compound **1**.

Compound **2**

**S 7.** ^1^H NMR spectrum of compound **2** (CD_3_OD, 600 MHz).

**S 8.** ^13^C NMR spectrum of compound **2** (CD_3_OD, 150 MHz).

**S 9.** HMBC spectrum of compound **2**.

**S 10.** COSY spectrum of compound **2**.

**S 11.** HSQC spectrum of compound **2**.

**S 12.** ROESY spectrum of compound **2**

Compound **3**

**S 13.** ^1^H NMR spectrum of compound **3** (C_5_D_5_N, 600 MHz).

**S 14.** ^13^C NMR spectrum of compound **3** (C_5_D_5_N, 150 MHz).

**S 15.** HMBC spectrum of compound **3**

**S 16.** COSY spectrum of compound **3**

**S 17.** HSQC spectrum of compound **3**

**S 18.** ROESY spectrum of compound **3**
